# A new therapeutic strategy for lung tissue injury induced by influenza with CR2 targeting complement inhibitior

**DOI:** 10.1186/1743-422X-7-30

**Published:** 2010-02-09

**Authors:** Chuanfu Zhang, Yuanyong Xu, Leili Jia, Yutao Yang, Yong Wang, Yansong Sun, Liuyu Huang, Fei Qiao, Stephen Tomlinson, Xuelin Liu, Yusen Zhou, Hongbin Song

**Affiliations:** 1 Institute of Disease Control and Prevention, Academy of Military Medical Science, Beijing 100071, China; 2 Beijing Institute for Neuroscience, Capital Medical University, Beijing 100069, China; 3 Department of Microbiology and Immunology, Medical University of South Carolina, Charleston, South Carolina 29425, USA; 4 State Key Laboratory of Pathogen and Biosecurity, Beijing Institute of Microbiology and Epidemiology, Beijing 100071, China

## Abstract

**Background:**

Influenza is a respiratory disease that seriously threatens human health. In fact, influenza virus itself does not make critical contribution to mortality induced by influenza, but "cytokine storm" produced by the excessive immune response triggered by the virus can result in inflammatory reaction of lung tissues and fatal lung tissue injury, and thus increase influenza mortality. Therefore, besides antiviral drugs, immunosuppression drugs should also be included in infection treatment.

**Presentation of the hypothesis:**

Complement is the center of inflammatory reaction. If complement system is over activated, the body will have strong inflammatory reaction or tissue injury, resulting in pathological process. Many studies have proved that, inflammatory injury of lung tissues caused by influenza virus is closely related to complement activation. Therefore, inhibiting complement activation can significantly reduce inflammatory injury in lung tissues. As complement is both a physiological defense and pathological damage medium, systematic inhibition may result in side effects including infection. Therefore, we design targeting complement inhibitors for complement activation sites, i.e. with CR2 as targeting vector, complement inhibitors like CD59 and Crry are targeted to inflammatory sites to specially inhibit the complement activation in local injury, thus local inflammatory reaction is inhibited.

**Testing the hypothesis:**

CR2-CD59 and CR2-Crry targeting complement inhibitors are fusion-expressed, and their biological activity is examined via in *vivo *and in vitro tests. CR2 targeting complement inhibitors are used to treat mouse influenza viral pneumonia model, with PBS treatment group as the control. The survival and lung tissue injury of the mice is observed and the effect of CR2 targeting complement inhibitors on pneumonia induced by influenza virus is evaluated.

**Implications of the hypothesis:**

CR2 targeting complement inhibitors are expected to be ideal drugs for viral pneumonia.

## Background

Influenza is an acute infectious disease caused by influenza virus, with respiratory damage as main outcome. It is epidemiologically characterized as rapid prevalence, wide dissemination, acute incidence and huge hazard, and is one of diseases that seriously threaten human health. A report by World Health Organization shows that there are 3-5 million severe influenza cases and 250,000-500,000 mortality every year [1]. Influenza pandemias happened for four times in the 20^th ^century. The Spanish flu in 1918 was the most serious one. It claimed 50 million lives at least, even more than the mortality in Fist World War [2]. More than 10,000 people died of H1N1 flu in 2009 [3]. Influenza produces a large number of morbidity and mortality, and also results in great economic loss and social burden.

The over reaction of immune system is an important reason for patient mortality. Oda T et al. pointed out in 1989 that symptoms of influenza are inflammatory injury as a result of immune activation by influenza virus, instead of being directly induced by influenza virus [4]. Immune system is activated in case of invasion by influenza virus. Studies have shown that when influenza virus invades human cells, cytokines and chemotatic factors are stimulated to produce many inflammatory proteins, which helps to defense virus [5-8]. Chemotatic factors and cytokines are the messengers of immune system, and play an important role in coordination and regulation of immune response. When influenza virus enters lung tissues, the immune system will lose control and make over reaction by releasing too many cytokines like "cytokines storm" [9,10]. Immune system running out of control will induce severe inflammation, and results in indirect hazard, which may induce inflammation again, damage the lung, and finally result in fatal pneumonia and acute respiratory tract infection syndromes. This indicates that influenza patients require both antiviral drugs and immunosuppression drugs [10]. Studies have shown that inflammatory injury of lung tissues is the main fatal reason for influenza A (H1N1) and bird flu, SARS, septicemia, aspiration pneumonia and liver infection induced by anthrax Bacillus as well [10-13].

## Presentation of the hypothesis

### Complement is the center of inflammatory reaction

Complement is an important and conservative system for natural immune, and provides pathways for rapid and effective elimination of invasive micro-organisms [14,15]. It is a "bridge" between natural immune and acquired immune. Besides direct immune mechanism, complement can also release many types of small molecular fragments which have broad biological effects, such as chemotaxis of neutrophils and lymphocytes, phagocytosis, and participation in regulating immune response of cells and body fluid. In addition, Complement system is also an important medium for inflammation and immune reaction, and poses great potential threat to the body. If complement system is over activated, many complement components will be consumed, and reduce the anti-infection ability of the body; many active substances derived from the activation will induce severe inflammatory reaction or tissue injury, resulting in pathological process [16]. For example, complement activation can produce inflammatory media including C2a, C3a, C4a and C5a. C2a has kinin-like function, and can expand small vessels and improve permeability; C3a, C4a and C5a have anaphylatoxin function, and can degranulate mast cells and basophils, release vasoactive mediators and induce inflammatory reaction; C3a, C5a and C5b67 have chemotaxis function, and can attract inflammatory cells to concentrate and migrate toward the inflammatory region activated by the complement, and thus increase inflammatory reaction.

CR2 is the central molecule for the immune response regulation by complement system. Split products of C3 molecules includes C3dg, iC3b, C3d and C3b, which are deposited on the activating cell surface and are the specific ligands for CR2 molecules. So CR2 is a good choice as a tarteting vector for delivery of complement inhibitors such as Crry and CD59 to sites of inflammation induced by complement activation. Many studies have indicated that CR2 targeting complement inhibitors can significantly mitigate inflammatory reaction in local sites [17,18]. CD59 and Crry are important complement regulatory protein and the ideal complement inhibitor. CD59 can interfere the combination of C7, C8 with C5b-6 complex and inhibite the formation of membrane attack complex, MAC. Crry can block the complement activation by inhibite the activity of C3/C5 convertase.

### Influenza viral lung injury and complement activation

Many studies have proved that excessive inflammatory injury in lung tissues induced by influenza virus infection is closely related to complement activation. Complement activation can affect influenza virus-specific immune response in the lung [19,20]. After being infected by influenza virus, C3-deficient mice see significant decrease of T-cell reaction, and complement activation plays an important role in T-cell activation or recruitment [21,22]. Martin has found that C3a and C5a can induce neutrophil migration in the lung infected by influenza virus [23]. All the above studies show that complement activation following influenza virus infection can significantly influence pulmonary infiltration and lung injury degree. Hohenthal U and Nuutila J found that complement receptors have strong expression in influenza viral pneumonia [24,25]. Kase T found that human MBL can directly or indirectly remove influenza virus particles and inhibit viral transmission through complement activation and opsonization [26]. Through coupling with influenza antigen HA, C3d can increase the level of anti-influenza virus HA antibody, reduce the activation threshold of B-cell and improve the intensity of immune response [27,28]. M. Paula Longhi et al. found that CD59a-deficient mice (Cd59a(-/-)) inflected with influenza virus have more serious pneumonia than wild-type, with more significant pulmonary hemorrhage and leukocytic infiltrate, neutrophil and lymphocyte aggregation, lung cell fibrosis and CD4^+ ^T-cell activation; after injection of complement inhibitors, Cd59a(-/-) mice have improved lung inflammatory reaction and significant neutrophil infiltration decrease [29].

### Hypothesis

The above studies indicate that through inhibiting complement activation, excessive inflammatory reaction in lung tissues induced by influenza can be inhibited, and as a result, lung tissue injury can be mitigated and the mortality can be reduced. As complement is both a physiological defense and a pathological damage medium, it functions as a double-edged sword. Systematic complement inhibition may result in potential side effects including infection. Therefore, we design targeting complement inhibitors for complement activation sites with CR2 as targeting vector, complement inhibitors like CD59 and Crry are targeted to inflammatory sites to specially inhibit the complement activation in the local injury, thus local inflammatory reaction is inhibited, without side effects caused by systematic inhibition.

## Testing the hypothesis

CR2 gene was respectively linked to genes of complement inhibitors including CD59 and Crry (CR2-CD59, CR2-Crry), and then is fusion expressed in CHO cells and purified from culture supernatant by affinity chromatography. Biological activity of CR2 targeting complement inhibitors is examined with *in vivo *and in *vitro *tests. BALB/c mice are applied to inhale mouse lung-adapted virulent strain of H1N1 influenza A virus (A/fm/1/47) via nose to duplicate influenza pneumonia model in mice. The mice are then treated with CR2 targeting complement inhibitors, with PBS treatment group as the control. Final work is to observe the survival and lung tissue injury of the mice, and evaluate the effect of CR2 targeting complement inhibitor on influenza viral pneumonia.

## Implication of the hypothesis

An effective CR2 targeting complement inhibitor can reduce the mortality, significantly improve clinical symptoms (decreased weight, lung index and hemagglutination titer) and lung tissue inflammatory injury of virus-infected model group. Therefore, CR2 targeting complement inhibitor is expected to be an ideal drug for viral pneumonia.

## Competing interests

The authors declare that they have no competing interests.

## Authors' contributions

CFZ, LYH and HBS prepared the paper. YTY, XLL, YSS, YYX, FQ, Stephen T, YSZ, XLL and LLJ participated in developing the hypothesis and collaborated in writing and reviewing of the article. All authors read and approved the final manuscript.

## References

[B1] Kamps BS Hoffmann C Preiser W Influenza Report 2006 Flying Publisher 18

[B2] Taubenberger J Morens D The 1918 Influenza: the mother of all pandemics Emerg Infect Dis 2006 12 15 22 16494711 10.3201/eid1201.050979 PMC3291398

[B3] http://www.who.int/csr/don/2009_12_23/en/index.html

[B4] Oda T Akaike T Hamamoto T Suzuki F Hirano T Maeda H Oxygen radicals in influenza-induced pathogenesis and treatment with pyran polymer-conjugated SOD Science 1989 26 974 976 10.1126/science.2543070 2543070

[B5] Woo PC Tung ET Chan KH Lau CC Lau SK Yuen KY Cytokine Profiles Induced by the Novel Swine-Origin Influenza A/H1N1 Virus: Implications for Treatment Strategies J Infect Dis 2010 201 346 353 10.1086/649785 20030555 PMC7202468

[B6] Deng R Lu M Korteweg C Gao Z McNutt MA Ye J Zhang T Gu J Distinctly different expression of cytokines and chemokines in the lungs of two H5N1 avian influenza patients J Pathol 2008 216 328 36 10.1002/path.2417 18788084

[B7] Us D Cytokine storm in avian influenza Mikrobiyol Bul 2008 42 365 80 18697437

[B8] Zheng BJ Chan KW Lin YP Zhao GY Chan C Zhang HJ Chen HL Wong SS Lau SK 8 Woo PC Chan KH Jin DY Yuen KY Delayed antiviral plus immunomodulator treatment still reduces mortality in mice infected by high inoculum of influenza A/H5N1 virus Proc Natl Acad Sci 2008 105 8091 8096 10.1073/pnas.0711942105 18523003 PMC2430364

[B9] Allen IC Scull MA Moore CB Holl EK McElvania-TeKippe E Taxman DJ Guthrie EH Pickles RJ Ting JP The NLRP3 inflammasome mediates in vivo innate immunity to influenza A virus through recognition of viral RNA Immunity 2009 30 556 565 10.1016/j.immuni.2009.02.005 19362020 PMC2803103

[B10] Kobasa D Jones SM Shinya K Kash JC Copps J Ebihara H Hatta Y Kim JH Halfmann P Hatta M Feldmann F Alimonti JB Fernando L Li Y Katze MG Feldmann H Kawaoka Y Aberrant innate immune response in lethal infection of macaques with the 1918 influenza virus Nature 2007 445 319 323 10.1038/nature05495 17230189

[B11] Nicholls J Peiris M Good ACE, bad ACE do battle in lung injury, SARS Nat Med 2005 11 821 822 10.1038/nm0805-821 16079870 PMC7095949

[B12] Imai Y Kuba K Rao S Huan Y Guo F Guan B Yang P Sarao R Wada T Leong-Poi H Crackower MA Fukamizu A Hui CC Hein L Uhlig S Slutsky AS Jiang C Penninger JM Angiotensin-converting enzyme 2 protects from severe acute lung failure Nature 2005 436 112 116 10.1038/nature03712 16001071 PMC7094998

[B13] Mossel EC Huang C Narayanan K Makino S Tesh RB Peters CJ Exogenous ACE2 expression allows refractory cell lines to support severe acute respiratory syndrome coronavirus replication J Virol 2005 79 3846 3850 10.1128/JVI.79.6.3846-3850.2005 15731278 PMC1075706

[B14] Walport MJ Complement. Second of two parts N Engl J Med 2001 344 1140 1144 10.1056/NEJM200104123441506 11297706

[B15] Stoiber H Banki Z Wilflingseder D Dierich MP Complement-HIV interactions during all steps of viral pathogenesis Vaccine 2008 26 3046 3054 10.1016/j.vaccine.2007.12.003 18191309

[B16] Blach-Olszewska1 Z Leszek J Mechanisms of over-activated innate immune system regulation in autoimmune and neurodegenerative disorders Neuropsychiatr Dis Treat 2007 3 365 372 19300567 PMC2654796

[B17] Song HB He C Knaak C Guthridge MJ Holers VM Tomlinson S Complement receptor 2-mediated targeting of complement inhibitors to sites of complement activation J Clin Invest 2003 111 1875 1885 12813023 10.1172/JCI17348 PMC161422

[B18] Song HB Qiao F Atkinson C Holers VM Tomlinson S A complement C3 inhibitor specifically targeted to sites of complement activation effectively ameliorates collagen-induced arthritis in DBA/1J mice J Immunol 2007 179 7860 7867 18025232 10.4049/jimmunol.179.11.7860

[B19] Kopf M Abel B Gallimore A Carroll M Bachmann MF Complement component C3 promotes T-cell priming and lung migration to control acute influenza virus infection Nat Immunol 2002 8 373 378 10.1038/nm0402-373 11927943

[B20] Kim AH Dimitriou ID Holland MC Mastellos D Mueller YM Altman JD Lambris JD Katsikis PD Complement C5a receptor is essential for the optimal generation of antiviral CD8^+ ^T cell responses J Immunol 2004 173 2524 2529 15294968 10.4049/jimmunol.173.4.2524

[B21] Mulligan M Watson S Fennie C Ward P Protective effects of selectin chimeras in neutrophil-mediated lung injury J Immunol 1993 151 6410 6417 7504020

[B22] Kopf M Abel B Gallimore A Carroll M Bachmann MF Complement component C3 promotes T-cell priming and lung migration to control acute influenza virus infection Nat Med 2002 8 373 378 10.1038/nm0402-373 11927943

[B23] Martin U Bock D Arseniev L Tornetta MA Ames RS Bautsch W Köhl J Ganser A Klos A The human C3a receptor is expressed on neutrophils and monocytes, but not on Bor Tlymphocytes J Exp Med 1997 186 199 207 10.1084/jem.186.2.199 9221749 PMC2198980

[B24] Hohenthal U Nuutila J Lilius EM Laitinen I Nikoskelainen J Kotilainen P Measurement of complement receptor 1 on neutrophils in bacterial and viral pneumonia BMC Infect Dis 2006 24 6 11 10.1186/1471-2334-6-11 PMC1397848 16433910

[B25] Nuutila J Hohenthal U Laitinen I Kotilainen P Rajamäki A Nikoskelainen J Lilius EM Quantitative analysis of complement receptors, CR1 (CD35) and CR3 (CD11b), on neutrophils improves distinction between bacterial and viral infections in febrile patients: comparison with standard clinical laboratory data J Immunol Methods 2006 315 191 201 10.1016/j.jim.2006.07.021 16970963

[B26] Kase T Suzuki Y Kawai T Sakamoto T Ohtani K Eda S Maeda A Okuno Y Kurimura T Wakamiya N Human mannan-binding lectin inhibits the infection of influenza A virus without complement Immunology 1999 97 385 392 10.1046/j.1365-2567.1999.00781.x 10447758 PMC2326860

[B27] Ross TM Xu Y Bright RA Robinson HL C3d enhancement of antibodies to hemagglutinin accelerates protection against influenza virus challenge Nat Immunol 2000 1 127 131 10.1038/77802 11248804 PMC1635154

[B28] Watanabe I Ross TM Tamura S Ichinohe T Ito S Takahashi H Sawa H Chiba J Kurata T Sata T Hasegawa H Protection against influenza virus infection by intranasal administration of C3d-fused hemagglutinin Vaccine 2003 21 4532 4538 10.1016/S0264-410X(03)00510-3 14575764

[B29] Longhi MP Williams A Matthew Wise Paul Morgan B Gallimore Awen CD59a deficiency exacerbates influenza-induced lung inflammation through complement-dependent and-independent mechanisms Eur J Immunol 2007 37 1266 1274 10.1002/eji.200636755 17429844 PMC2435422

